# Preoperative Synbiotic Supplementation and Postoperative Outcomes in Elective GI Surgery: A Double-blinded, Placebo-controlled Trial: Short title: Synbiotics and Elective GI Surgery

**DOI:** 10.31661/gmj.v15i.3580

**Published:** 2026-06-08

**Authors:** Pourya Adibi, Ahmad Reza Karimi, Mehrdad Sayadinia

**Affiliations:** ^1^ Department of Anesthesiology, Critical Care and Pain Management Research Center, Faculty of Medicine, Hormozgan University of Medical Sciences, Bandar Abbas, Iran; ^2^ Department of Surgery, Faculty of Medicine, Hormozgan University of Medical Sciences, Bandar Abbas, Iran; ^3^ Faculty of Medicine, Hormozgan University of Medical Sciences, Bandar Abbas, Iran

**Keywords:** Synbiotics, Complication, Gastrointestinal, Trial, Microbiota

## Abstract

**Background:**

Surgery harms the gastrointestinal (GI) tract barrier and changes intestinal microbiota composition, disrupting the balance of gut flora. Synbiotics, which are mixtures of beneficial bacteria and chemicals that promote gut flora growth, are particularly important for recovery after GI surgery. We aimed to assess the effect of consumption of synbiotics prior to the gastrointestinal surgeries on complications.

**Materials and Methods:**

40 patients who planned to undergo elective gastrointestinal surgery randomized in a double-blinded, placebo-controlled, phase 3 trial to a single dose perioperative and seven-day postoperative intervention with a synbiotic, LactoCare®, or placebo provided by knowledge-based spin-off company, Zist Takhmir. Randomisation was performed by a computer algorithm.

**Results:**

42.5% of participants were men. Nausea and vomiting occurred in eight patients in the placebo group, and seven patients in the synbiotic group, and the two groups were not statistically significant (P0.99). Other complications, such as (surgical site infection, fever, surgical site infection, pneumonia, and anastomosis site leakage) were not observed in the placebo or synbiotic groups. In assessing variables dependent on time, the mean time to start oral feeding in the synbiotic group was 32.35±28.84 hours after surgery, and in the placebo group was 28.45±24.91 hours (P=0.796). The first gas passing time was 13.00±15.81 hours in the synbiotic group and 9.65±13.90 hours in the placebo group after surgery(P=0.406). The hospitalisation length of patients in the synbiotic group was 2.6±1.27 days, and for patients in the placebo group was 2.35±1.08 days(P=0.42).

**Conclusion:**

Administration of synbiotics as a single dose before surgery and seven days postoperatively did not have a significant effect on infectious complications and time-dependent variables in patients with abdominal surgery.

## Introduction

Probiotics are beneficial living microorganisms [1]. Prebiotics are non-digestible food components, that encourage the development of a healthy gut microbiome [2]. Synbiotics are the combination of one or more probiotics and one or more prebiotics [1]. The gastrointestinal tract is the main residence of microorganisms called "intestinal microbiota." The symbiosis between humans and intestinal microbiota is necessary for health. Dysbiosis is associated with several health problems, such as colonic inflammation, neurodegenerative diseases, metabolic disorders, and cardiovascular conditions, as well as obesity-related illnesses and certain types of cancer [3][4][5][6][7].

Some literature found that administering synbiotics was related to lower surgery-related complications [8] and could contribute to the promotion of gastrointestinal function recovery after GI cancer surgery [9]. Patients planning to undergo GI surgery receive preoperational antibiotics, which can lead to changes in the gut microbiome [10][11]. According to recent findings, synbiotics could decrease the risk of infection after abdominal surgery and promote GI motility [12][13]. Studies have shown conflicting results on using synbiotics as a strategy to promote recovery of GI function and post-surgical complications [14][15]. Synbiotics in elective surgery patients without risk factors such as immunosuppression or critical disease are considered safe [16].

Surgery alone harms the GI tract, a barrier preventing bacterial adhesion [17]. Furthermore, significant alterations in the local microenvironment during gastrointestinal surgery profoundly impact the intestinal microbiome's diversity. The sudden exposure of the intestinal lumen to oxygen, normally a hypoxic environment, precipitates tissue hypoxia due to the disruption of local perfusion. Consequently, the interplay between obligatory and facultative anaerobic shifts. Disruption of the gut's microbial balance can lead to postoperative sepsis [17].

Many studies showed that during the postoperative period, harmful bacteria such as Pseudomonas, Staphylococcus, and Enterococcus, which can cause postoperative infection, have increased [18]. The goal of this study was to investigate the potential benefits of preoperative administration of synbiotics on reducing postoperative complications in patients undergoing elective gastrointestinal surgery. This objective is well-justified, as surgery can disrupt the gut microbiota and increase the risk of complications such as infections and delayed recovery. The study's focus on a specific and well-defined patient population [elective GI surgery patients] and the use of a randomized, double-blinded, placebo-controlled design provide a strong foundation for assessing the efficacy of synbiotics in this context. Additionally, the study's evaluation of both infectious complications and time-dependent variables (such as time to start oral feeding and hospital stay) provides information about the potential benefits of synbiotics in this setting.

## Materials and Methods

This randomized, double-blinded, placebo-controlled clinical trial. The Iranian Registry of Clinical Trials (IRCT) approved the research protocol under the registration number IRCT20210804052083N1. The study sample acquisition was held from March 21, 2022, to May 20, 2022, in the tertiary public hospital of Shahid Mohammadi, Bandar Abbas, Iran.

A sample size calculation was performed to determine the required number of patients for the study [19]. Based on a predicted incidence of 57.1% in the placebo group and 19.0% in the intervention group for bacteraemia as the complication, and utilizing a two-tailed test with a significance level of 0.05, our calculations indicated that a sample size of 20 participants per arm would yield approximately 80% statistical power to identify a significant disparity in the occurrence of postoperative bacteremia between the control and treatment cohorts.

The participants and study investigators remained blinded until statistical analysis was completed. All patients referred to the institution who were scheduled to undergo elective gastrointestinal surgery for any reason, such as colorectal and hepatobiliary cancers, gallbladder stones, etc. included. Patients with underlying chronic diseases of diabetes, coagulation disorders, congenital or acquired immunodeficiency, liver cirrhosis, renal failure, and acute pancreatitis were excluded.

Participants were randomly allocated to either the synbiotic or placebo group using a random number table, where each participant was assigned a unique random number and then allocated to a group based on a predetermined sequence of numbers. This process was repeated until all participants were allocated to a group. An unbiased third party, unaffiliated with patient recruitment or code assignment, utilized web-based software (https://www.sealedenvelope.com/) to generate random sequences through the permuted block randomization technique. Random allocation was performed in blocks of sizes 2 and 4, without stratification, to ensure equal distribution of participants.

Capsules containing synbiotics or placebo, completely identical, sealed, and packed by the pharmaceutical company labeled A or B, were provided to participants. Notably, the company was the only one that knew which product was synbiotic and which was a placebo. The principal investigator, the night before surgery, was given a capsule. Patients were instructed to take subsequent doses after oral administration after lunch and dinner for seven days.

The synbiotic used was LactoCare®, provided by a knowledge-based spin-off company, Zist Takhmir, a dietary supplement constituting 500 mg of Bifidobacterium lactis, Bifidobacterium breve, Bifidobacterium bifidum, Bifidobacterium longum, Lactobacillus acidophilus, Lactobacillus bulgaricus, Lactobacillus casei, Lactobacillus helveticus, Lactobacillus plantarum, Lactobacillus rhamnosus, Streptococcus thermophilus, and Fructooligosaccharides (FOS). The placebo content used in the study was lactose monohydrate, talc, magnesium stearate, maltodextrin, silicon dioxide, microcrystalline cellulose, and sodium starch glycolate Capsules containing synbiotics or placebo were stored by the pharmaceutical company and provided to participants, who were instructed to take them orally at home, with one dose given by the principal investigator the night before surgery and subsequent doses taken by the patients at home after lunch and dinner for seven days.

During the intervention, the principal investigator called each patient daily to ensure they took their capsules. At the beginning of the intervention, baseline data of all participants was documented, including age, sex, body mass index (BMI), reason for surgery (cancer, cholecystitis, gall stones, etc.), and previous history of abdominal surgery.

The primary endpoint of this study to evaluate the effect of synbiotics on the incidence of postoperative complications, including nausea and vomiting, fever, SIRS (Systemic Inflammatory Response Syndrome), pneumonia, SSI (Surgical Site Infection), anastomotic leak, and organ failure (heart, liver, kidney) in patients undergoing surgery. Secondary objectives were to assess the effect of synbiotics on the time to start feeding after surgery; To evaluate the effect of synbiotics on the time to first gas passing or defecation after surgery; To compare the length of hospital stay between patients receiving synbiotics and those receiving a placebo.

A systemic inflammatory reaction syndrome (SIRS) diagnosis requires the presence of at least two of the following criteria: (1) a core body temperature exceeding 38 degrees Celsius or falling below 36 degrees Celsius, (2) a respiratory frequency exceeding 20 breaths per minute, (3) a cardiac rate surpassing 90 beats per minute, and (4) an elevated or decreased white blood cell count, with values exceeding 12,000 cells per microliter or dropping below 4,000 cells per microliter. Pneumonia was defined as a treating physician diagnosis with new pulmonary infiltrates on chest imaging, fever, and/or leucocytosis. Surgery site infection (SSI) was defined as any purulent or serous drainage from the laparotomy incision that has been present for more than 24 hours and/or developed during hospitalization. breach in the intestinal wall at the site of surgical connection was classified as an anastomotic leakage.

Cardiac insufficiency was characterized by a left ventricular ejection fraction below the threshold of 40%, as determined by the attending physician's professional judgment. Impaired renal function was identified by an estimated glomerular filtration rate of less than 60 milliliters per minute per 1.73 square meters of body surface area. Hepatic impairment was indicated by elevated levels of either alanine transaminase (ALT) or aspartate transaminase (AST), exceeding the upper limit of the normal range, which was established at 40 units per liter.

The distribution of categorical data was compared using a chi-squared test or Fisher's exact test, as appropriate. Continuous variables were compared using independent t test. A P-value of lower than 0.05 was considered statistically significant using SPSS v.19.

## Results

**Figure-1:**
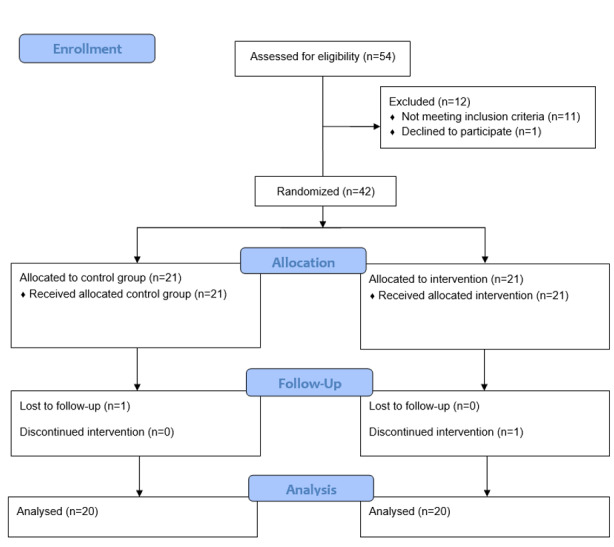
Figure 1.

**Table:** Table1. Characteristics at Inclusion of Participants in the Study

**Characteristic**	**Synbiotic group**	**Placebo group**	**P-value**
**Sex (n)**			
**Male**	8(47.1%)	9(52.9%)	0.749
**Female**	12(52.2%)	11(47.8%)	
**Age (y)**	48.05±13.93	44.55±10.53	0.409
**BMI(kg/m** **^2^** **)**	23.54±3.34	23.64±3.56	0.957
**Reason of surgery (n)**			
**Cholecystitis**	16(47.1%)	18(52.9%)	0.661
**cancer**	4(66.7%)	2(33.3%)	
**Past history of surgery(n) **	6(50%)	6(50%)	1

**Table:** Table 2. Time Dependent variables

**Variable**	**Synbiotic group**	**Placebo group**	**P-value**
**Time to start feeding(h)**	32.35±28.84	28.45±24.91	0.796
**Time to first gass passing or defecation(h) **	13.00±15.81	9.65±13.90	0.406
**Hospital stay(d)**	2.6±1.27	2.35±1.08	0.42

There was 1 lost to follow up in control group and 1 discontinued intervention in intervention group due to patients noncompliance to use the medication. Twenty subjects were evaluated in each group.

Table-1 presents the baseline characteristics of participants in the synbiotic and placebo groups, showing no significant differences in sex distribution (male: 47.1% vs. 52.9%, female: 52.2% vs. 47.8%, P=0.749), mean age (48.05 ± 13.93 vs. 44.55 ± 10.53 years, P=0.409), BMI (23.54 ± 3.34 vs. 23.64 ± 3.56 kg/m², P=0.957), reason for surgery (cholecystitis: 47.1% vs. 52.9%, cancer: 66.7% vs. 33.3%, P=0.661), or past surgical history (50% vs. 50%, P=1).

Nausea and vomiting occurred in 8 patients in the placebo group, and seven patients in the synbiotic group, and the two groups were not statistically significant (P>0.99). Other complications, such as (surgical site infection, 30-day mortality, fever, surgical site pneumonia, and anastomosis site leakage) were not observed in the placebo or synbiotic groups. Therefore, the relationship between the effect of synbiotics on these complications after gastrointestinal surgery was not measurable.

In assessing variables dependent on time, the mean time to start oral feeding in the synbiotic group was 32.35± 28.84 hours after surgery. In the placebo group, it was 28.45±24.91 hours (P=0.796). The first gas passing time was 13.00±15.81 hours in the synbiotic group and 9.65±13.90 hours in the placebo group after surgery(P=0.406). The duration of hospital stay for patients in the synbiotic group was 2.6±1.27 days, and for patients in the placebo group, it was 2.35±1.08 days (P=0.42, Table-2). As a result, synbiotics have no significant effect on the duration of starting oral feeding, gas passing or defecation, or hospital stay after GI surgery (P>0.05).

## Discussion

Our study showed that administration of synbiotics as a single dose before surgery and seven days postoperatively did not have a significant effect on infectious complications and time-dependent variables in patients with abdominal surgery. Our findings are consistent with a previous clinical trial by Franko et al. [20], which also showed that perioperative use of probiotics in abdominal surgery did not affect mortality and infection rate but was associated with a significantly higher readmission rate. Another study by Anderson et al. [21] found that two weeks of administration of synbiotics before elective abdominal surgery showed no significant differences in septic complications in placebo and synbiotic groups. In contrast to the mentioned research, some studies demonstrated conflicting results. Sugawara et al. [18] has demonstrated that pre-surgical oral supplementation with synbiotics can bolster the body's immune defenses, mitigate the severity of systemic inflammation following surgery, and foster a more favorable balance of gut microbiota. A systematic review and metanalysis of 34 studies carried out by Chowdhury et al. [12] in 2019 to determine the impact of probiotics or synbiotics on postoperative complications in adults undergoing elective abdominal surgery found a significant reduction in hospital stay and the incidence of postoperative infectious complications. However, the study reported no significant effect on mortality and non-infectious complications. Consistent with Chowdhury et al. [12] findings, another systematic review and metanalysis by Amitay et al. [22] in 2020 on the effect of perioperative use of synbiotics/probiotics on postoperative complications in patients with colorectal cancer undergo colorectal cancer resection, they found the association of use of synbiotics/probiotics with lower infection incidence and shorter length of hospital stay. This study demonstrated that administering oral synbiotics from one day before GI surgery to seven days postoperatively had a significant effect on diarrheal incidence, returning to normal gut function and first defecation, plus days of antibiotics use. The differences in the results of the present study and Franko et al. with meta-analyses and systematic reviews by Amitay et al. and Chowdhury et al. [12] can be primarily due to differences in the synbiotics and probiotics used. For example, in the present study, the synbiotic used included 11 strains of Lactobacillus, Bifidobacterium, and Streptococcus thermophile bacteria. Still, this number was less than five strains in most of the studies used in Amitay's research. Also, the number of synbiotics/probiotics colonies differs in different studies. Another reason for this difference in the results can be various study methods. In our study, the first dose of synbiotics/probiotics was used the night before the operation, while among the 16 articles used in the Amitay meta-analysis, only one study used the first dose the day before the operation, and other studies started on an average of 4-6 days before the operation. Moreover, in the study of Chowdhury et al. [12], out of 34 reviewed articles, in 28 studies, prophylaxis treatment was started. In 26 studies, the minimum time to start treatment was three days before surgery. Also, the duration of follow-up and postoperative treatment has been longer in other studies, which can be one of the reasons for the differences obtained in the mentioned studies.

While Fabiani and Lestari’s [22] evidence-based case report suggests that perioperative probiotics or synbiotics may aid gastrointestinal recovery, albeit without significantly reducing postoperative ileus we found no such benefits, possibly due to differences in intervention timing (single-dose vs. prolonged supplementation) or patient populations (general abdominal surgery vs. elective GI surgery). In another trial that sought preoperative symbiotic, Maher et al.’s study demonstrated that prolonged pre- and postoperative synbiotic supplementation enhanced CD8+ T cell infiltration, reduced pro-inflammatory cytokines, and lowered complication rates in pancreatic cancer patients [23]. So, the issue of single dose or prolonged administration of symbiotic should be re-evaluated in further research.

## Conclusion

In conclusion, administration of synbiotics as a single dose before surgery and seven days postoperatively did not significantly affect infectious complications, time to start oral feeding, first gas passing time, and duration of hospital stay in patients with abdominal surgery.

## Conflict of Interest

The authors affirm that there are no competing interests or conflicts of interest to report.

## AI Disclosure Statement

During the preparation of this manuscript, the authors used ChatGPT, OpenAI company for language editing, grammar improvement, and liboberry.com for reference management. After its use, the authors thoroughly reviewed, verified, and revised all AI-assisted content to ensure accuracy and originality. The authors take full responsibility for the integrity and final content of the published article.
